# Perceptions and practices on newborn care and managing complications at rural communities in Bangladesh: a qualitative study

**DOI:** 10.1186/s12887-021-02633-z

**Published:** 2021-04-09

**Authors:** Abu Sayeed Md. Abdullah, Koustuv Dalal, Masuma Yasmin, Gainel Ussatayeva, Abdul Halim, Animesh Biswas

**Affiliations:** 1 Centre for Injury Prevention and Research Bangladesh, Dhaka, Bangladesh; 2 Department of Public Health Science, School of Health Sciences, Mid Sweden University, Sundsvall, Sweden; 3 Department of Epidemiology, Biostatistics and EBM; Faculty of Medicine and Health Care, al-Farabi Kazakh National University, Almaty, Kazakhstan; 4 Kolkata, India; 5 Dhaka, Bangladesh

**Keywords:** Neonatal care, Neonatal complication, Perceptions, Practice, Bangladesh

## Abstract

**Background:**

Community misperception on newborn care and poor treatment of sick newborn attributes to neonatal death and illness severity. Misperceptions and malpractices regarding neonatal care and neonatal complications are the leading causes of neonatal deaths in Bangladesh. The study was conducted to explore neonatal care’s perceptions and practices and manage complications among Bangladesh’s rural communities.

**Methods:**

A qualitative study was conducted in Netrakona district of Bangladesh from April to June 2015. Three sub-districts (Upazilas) including Purbadhala, Durgapur and Atpara of Netrakona district were selected purposively. Five focus group discussions (FGDs) and twenty in-depth interviews (IDIs) were conducted in the rural community. Themes were identified through reading and re-reading the qualitative data and thematic analysis was performed.

**Results:**

Community people were far behind, regarding the knowledge of neonatal complications. Most of them felt that the complications occurred due to lack of care by the parents. Some believed that mothers did not follow the religious customs after delivery, which affected the newborns. Many of them followed the practice of bathing the newborns and cutting their hair immediately after birth. The community still preferred to receive traditional treatment from their community, usually from Kabiraj (traditional healer), village doctor, or traditional birth attendant. Families also refrained from seeking treatment from the health facilities during neonatal complications. Instead, they preferred to wait until the traditional healers or village doctors recommended transferring the newborn.

**Conclusions:**

Poor knowledge, beliefs and practices are the key barriers to ensure the quality of care for the newborns during complications. The communities still depend on traditional practices and the level of demand for facility care is low. Appropriate interventions focusing on these issues might improve the overall neonatal mortality in Bangladesh.

**Supplementary Information:**

The online version contains supplementary material available at 10.1186/s12887-021-02633-z.

## Background

Preterm birth complications, newborn infections and birth asphyxia are the leading causes of about 80% of the global neonatal deaths [1]. During the last three decades, newborns’ deaths have been almost halved (1990: 5 million, 2019: 2.4 million). In the year 2019, almost five newborns died every minute, where, three fourth of the deaths were within the first week. A significant proportion of these neonatal deaths occur in low-and middle-income countries [2]. A study conducted in India found that about half of the total neonates had the illness, among which half of them had one danger sign, out of which one fifth did not receive appropriate medical care, had local beliefs for neonatal danger signs and depended on unqualified providers for traditional treatment [3]. Bangladesh had newborns mortality of 56 per 1000 live births which rank eighth among the top ten countries with the highest numbers of newborns deaths in 2019 [2]. A recent nationwide study indicates that as Bangladesh has invested in achieving MDGs, facility-based deliveries had increased from 37% in 2014 to 50% in 2018. Postnatal checkup for the mothers (within 48 h of delivery) has increased from 34% in 2014 to 52% in 2018, indicating a significant improvement [4]. It is essential to mention here that Bangladesh has set a target of newborns mortality of 12 per 1000 live births by 2030. Bangladesh has planned for more comprehensive utilization of the community health workers including community health care providers (CHCP), Health Assistant (HA) and Family welfare assistant (FWAs) for better utilization of facility-based delivery and for improving newborn care practices [4]. The majority of the neonatal deaths occur in low and middle-income countries because of lack of access to proper neonatal care, as the bulk of the deliveries are conducted at home. However, in some cases, the deaths occur even after deliveries at facilities because regular neonatal care is undertaken soon after being discharged from the health facilities. Policymakers should focus on the implementation of intense interventions, in order to improve the neonatal care practices and to reduce the neonatal deaths in the community [5].

Many misperceptions and malpractices persist in the community, which ultimately lead to neonatal deaths. Most of the mothers had started breastfeeding on the third day after delivery. Most of the caregivers had fever, irritability, weakness, vomiting and diarrhoea, which are danger signs of neonatal morbidity [6]. Although acute respiratory illness (ARI) is one of the major causes of neonatal deaths in Bangladesh, most mothers could not recognize these danger signs. Instead, they sometimes believed it as the influence of evil and depended on spiritual healers, which ultimately delayed proper treatment [7]. Only 71% of the mothers practised breastfeeding on the first day of birth, about 70% exclusively breastfed during the neonatal period [8]. Neonatal care perceptions and practices include growth monitoring and appropriate home care, disease prevention activities, and seeking care during complications. However, these effective practices are still limited [9]. Provision of proper antenatal care, adoption of appropriate family planning methods, and prevention of physical violence and prematurity could bring about a considerable reduction in neonatal mortality [10]. Maternal recognition of neonatal complications at home is low at the community level, which is a significant factor standing in the way of the betterment of neonatal health. Therefore, essential interventions must be undertaken for the identification of the neonatal complications by mothers at home. Also, the community health care providers’ routine checkup might play an essential role in improving the care-seeking behaviour during neonatal complications [11]. The caregivers and health care providers need to identify the danger signs in sick neonates and seek the appropriate level of health care for the neonates. This would bring about a considerable reduction in neonatal mortality, one of the key indicators to assess the public health situation [6].

Many factors, such as non-availability of health care services, low quality, non-affordability, non-accessibility, transport costs, the dearth of knowledge regarding care-seeking, and cultural perceptions and practices are responsible for poor health care seeking for neonates [12]. The present study explores the perceptions and practices regarding neonatal care and health-seeking practices for managing neonatal complications among the rural communities in Bangladesh. Our study findings also intended to address the policy decisions regarding interventions to improve neonatal health by better understanding community perceptions and practices.

## Methods

A qualitative study was conducted in Netrakona district of Bangladesh from April to June 2015. Three sub-districts (Upazilas) including Purbadhala, Durgapur and Atpara were selected purposively, where five focus group discussions (FGDs) and twenty in-depth interviews (IDIs) were conducted. The individuals of the selected groups, aged above the range of 18 years, who were willing to participate, were selected to be the respondents in the study.

For FGDs, we chose groups of pregnant mothers; delivered mothers (delivered a live baby in last 3 months), female guardians of pregnant or recently delivered mothers including mother and mother-in-law, male guardians of pregnant or recently delivered mothers including husband, father and father-in-law and traditional birth attendant of that specific community. Each selected group consisted of six to eight participants (Table 1).

**Table 1:** List of Participants in the qualitative study

Qualitative instruments	Age range	Participants
FGD (*n* = 5) [Participants- 36]	18–50 years	Pregnant mothers [7] Mothers who delivered recently [8] Female guardians of pregnant/ recently delivered mothers [8] Male guardians of pregnant/ recently delivered mothers [7] Traditional Birth Attendants [6]
IDI (*n* = 20)	19–35 years	Mothers of deceased neonates [10] Fathers of deceased neonates [10]

The study found that 118 neonatal deaths had occurred from January to March 2015 in the three selected Upazilas. For in-depth interviews, the participants were chosen from these families where neonatal deaths had occurred. IDIs were performed with the families’ male and female guardians to understand their neonatal care and practices and health care seeking behaviour during neonatal complications. All IDIs were conducted following the guidelines for a face-to-face interview at the household level. A total of 46 participants were included in five FGDs and 20 IDIs. Thirty-six of the respondents participated in the five FGDs and 20 in the IDIs (Table 1).

Two trained research officers (anthropologists) were assigned to collect the data from the field. Formal training was provided; guidelines were pre-tested before data collection. During FGDs, one research officer facilitated the discussion, whereas, the other researcher took important notes. The objectives of the research were clearly demonstrated to the respondents before conducting the interviews. Rights to withdraw from the study was informed to the respondents before the study. Written consent was taken from each of the respondents before the interview or FGD.

The qualitative research guidelines (appended at the end of the manuscript) were developed by the trained research officers inconsulation with the experienced researchers. After pre-testing necessary changes were made and then used in the final phase of data collection. Different prompts/ probes were used during qualitative data collection (Table 2).

**Table 2:** Content of the focus-group discussion and In-depth interview

Area of discussion	Types of Prompts used
Perceptions of neonatal care and complications in the community	The idea about neonatal care and complication? Where and from whom got ideas about neonatal complication? Why did the responder not get appropriate ideas on neonatal care?
Practices regarding neonatal complications in the community	What community practices persist for neonatal care? What preparations are undertaken during neonatal complications? Where and from whom received treatment during neonatal complications? What are the social and familial barriers in the community, hindering the practice of proper neonatal care and health care seeking for treatment of the neonates?

The field team worked under the guidance of the Principal Investigator (PI), responsible for providing technical assistance in designing the study, data collection to some extent, analysis of information and writing the report. PI regularly discussed with the experienced research team members. The research team members were primarily responsible for designing and undertaking the survey and quality control, where PI took the lead role. The limitations of the study were the small sample size as well as the small study area.

The trained anthropologists conducted the IDIs and FGDs and transcribed them in the native Bengali language, based on audio-recordings and hand notes. Later, Bengali transcripts were translated into English. The PI and another experienced researcher randomly selected transcripts, reviewed and assured the quality. Both the PI and the experienced researcher were bi-lingual (Bengali and English). We used peer debriefing for maintaining data reliability. Selective coding was conducted from initial open codes and then themes were identified through reading and re-reading the qualitative data [13, 14]. Finally, the used thematic analysis for the study.

The national ethical review committee of the Centre for Injury Prevention and Research, Bangladesh (CIPRB) has approved this study. Written consents were received from each of the respondents before the IDIs and FGDs.

## Results

The majority of the participants, especially the female and male guardians and the traditional birth attendants, were found to have belief in myths and superstitions regarding neonatal complications, having no specific idea about neonatal care. Moreover, they adopted traditional practices to deal with neonatal complications. Majority of them thought that the neonatal complications occurred due to some past sins committed by the parents. It was firmly believed that such complications were bound to follow if the mother did not follow the religious rules and rituals.

After the neonates’ birth, the community people followed the practice of shaving their hair immediately and bathing them within a day of birth, because they believed that the babies were born with unclean liquids and dirty layers covering their bodies should be cleaned immediately with water. The study revealed that the husbands did not give neonatal care much importance and preferred to depend on traditional practices, instead of seeking treatment from qualified health care providers. They mainly depended on traditional birth attendants and village doctors for treatment of their neonates. Also, some of them depended on the traditional healers (Kabiraj) for the treatment of neonates. However, the groups of pregnant mothers and recently delivered mothers had clear ideas regarding neonatal care after birth and treatment during complications, but they could not practice this properly due to family and societal barriers. Some IDI participants could realize the importance of accurate practices during complications after the neonatal deaths that occurred in their families.

### Perceptions about neonatal care and complications

Most male and female guardians indicated to adopt traditional perceptions regarding neonatal care and complications. Although they had heard about the ideal rearing process from the health care providers in courtyard meetings, they firmly believed the traditional ideas. They preferred to follow the practices of bathing the neonates and shaving their hair immediately after birth. Moreover, due to their deep-rooted belief that colostrum is harmful to babies, they preferred not to provide it to the neonates 3 days after birth. They also thought that the neonatal complications occurred when the parents disobeyed the religious rules. However, pregnant mothers and recently delivered mothers had actual perceptions regarding neonatal care and complications.

During FGD, one of the traditional birth attendants said, *“We take care of lots of babies and learn from our experiences. Sometimes, the neonates' skin becomes discoloured like red and blue, with the complication of convulsions after birth. We believe that the baby is caught by the Takri and is difficult to cure."*

In IDI, one of the female guardians said, *“During our time, there was no problem with babies even if they were taken care of insincerely, but now the babies are born coughing and sneezing with pneumonia. Jaundice also occurs in the mother's womb. Much more money is required for their treatment in the hospital."*

During FGD, one recently delivered mother said, *“A baby may have lots of problems after birth, such as cold, navel infection, pneumonia and tetanus. They may have navel infection, like swelling or red blister on the body. I watched these on television in an educational program about maternal and neonatal health. I have also seen some problems with our neighbours.”*

In FGD, a female guardian stated, *“When I was pregnant many years back, nothing bad happened. However, I came to know that, nowadays, mothers should take more rest and babies should not take a bath before seven days. Because of these practices, many babies are dying. If mothers practised like we did many years back, nothing would happen.”*

**Table:** 

**Perception of the community**	
➢ Believe in myths and superstitions	
➢ Lack of knowledge regarding neonatal danger signs	
➢ Lack of perceptions regarding quality newborn care	
➢ Neonatal complications occur when parents disobey religious rules.	
➢ Neonatal deaths occur due to misfortune.	

### Practices during neonatal care and complications

The group of female guardians were found to practice traditional methods for treating the neonates during complications. They mainly depended on the Kabiraj or traditional healers for immediate treatment. If the traditional healers were unable to cure the neonates, they preferred to seek help from the traditional birth attendants and the village doctors. Lastly, they went to the health facilities for treatment of the neonates. They followed the traditional practice of applying mustard oil on the umbilicus of the neonates for healing purpose. They also preferred to bath the babies immediately after birth and shaving of hair for proper cleaning. Also, they even advised the mothers to remove the colostrum before providing milk to the babies. It was also a common practice to feed the babies, honey, before providing milk. The groups of male guardians and husbands believed that neonatal care was the women’s responsibility in the family. They were also not actively involved in emergency treatment during neonatal complications.

During FGD, one of the female guardians said, *“We worked a lot during pregnancy, but nowadays, the women always take rest. That is why the neonatal complications occur in the mother’s womb. We used to bathe our babies immediately after birth, but no problem used to occur. Nowadays, a new rule is followed: the baby should not be allowed to bathe before seven days of birth. After birth, we feed honey to the baby at first."*

During IDI, one of the female guardians said, *“I heard that there are two types of pneumonia, one occurs from hot and the other occurs from cold. We did not practice to provide breast milk to baby up to three days of birth, as we did not know about the importance of colostrum. However, nowadays, mothers provide the first colostrum to babies.”*

During FGD, one of the recently delivered mothers said, *“My mother arranged clean clothes and blankets before my delivery and she cleaned my baby with the dry cloth immediately after birth. I also provided breast milk to my baby within one hour of delivery. I did not shave my baby's hair as pneumonia might occur due to the baby's baldness. Moreover, I did not bathe him for the first three days after birth."*

During IDI, a female guardian mentioned, *“Neonatal complications usually occur when the parents disobey the religious rules and in such cases, it is difficult to cure the baby.”*

**Table:** 

**Practices of the community**	
➢ Dependency on traditional healers and unskilled providers	
➢ Ignorance of male guardians on newborn care and complications	
➢ The practice of bathing the baby immediately after delivery	
➢ Application of mustard oil on the umbilicus for healing purpose	
➢ Delay in decision making and transport delay to reach facilities during neonatal complications	

## Discussion

The study found that the misperception and malpractices persisted in the rural communities for newborn care and health-seeking practices for managing neonatal complications. The present study revealed that most people in the community, including the mothers-in-law, fathers-in-law and traditional birth attendants still practised traditional neonatal care and had no accurate knowledge regarding neonatal complications. They followed the practice of bathing the infants immediately after birth and shaving their heads because they believed that skin and hairs are covered by dirty fluids. Moreover, they also applied mustard oil on the umbilicus of the newborns for healing purpose. Similar findings were indicated in several other studies [6, 15–18]. A study conducted in Zambia found that various traditional beliefs and practices regarding childbirth continued to exist along with the modern health care system, which contributed to the increased rate of maternal and perinatal morbidity and mortality in Zambia. The newborn was placed on the delivery mat until the expulsion of the placenta. The umbilical cord was cut with the help of the razor blade or sugar cane peel. Both the mother and the newborn were bathed and then they applied ash on the umbilical cord stump [19]. Another study conducted in the Dhaka slums, Bangladesh reported that a razor blade was used in 95% of the cases and a bamboo strip in 5% of the cases for cutting the umbilical cord. Only 13% of cases used a sterilized razor blade and 71% did not apply anything on the stump [20]. A study, which was conducted among the caregivers and health workers in northern India, found that locally prepared kajal (eye-liner) was applied to the eyes of the newborns and a sharp iron object was kept in close vicinity to them, in order to ward off evil eyes and bad omens [6].

The neonatal deaths are mainly caused by preterm birth, asphyxia, infections and congenital disabilities [2]. Every hour, 274 newborn deaths occur globally due to negligence, some of which have preventable causes [2]. The neonatal mortality rate (NMR) was 29.2 in-home care, 45.2 in community care and 43.5 in comparison arm per 1000 live births, in Bangladesh [21]. The current study found that the female guardians and the traditional birth attendants advised the mothers to remove the colostrum before providing milk to their babies, because of the deep-rooted belief that colostrum was harmful. Also, the practice of feeding honey to the newborns was widely prevalent. These improper practices, including delayed breastfeeding, removal of colostrum and providing pre-lacteal feeds, resulted in newborns’ ill health and neonatal complications. The previous study conducted in Bangladesh stated that it was common to delay breastfeeding about 3 days after childbirth because colostrum was considered harmful and poisonous. It was believed that feeding it to the babies could cause abdominal pain and diarrhoea in them, because of the evil spirits in colostrum [15].

A qualitative study conducted in rural Ghana found that most of the families’ male members had little or no physical contact with the newborns. Due to existing gender norms, men are bread-earners of the family and are expected to work outside the house. On the other hand, the women were expected to perform the household works, including childcare. Breastfeeding, cord care, bathing and keeping the baby warm are the mothers’ and the female guardians’ duty [22]. Similarly, the present study also indicated that the husbands and the male guardians believed that childcare was the women’s responsibility and hence, they preferred to take no part in it.

Moreover, many of the community people believed in superstitions and myths for such complications and allowed spiritual treatment of the neonates. They depended firstly on Kabiraj or traditional healers, secondly on traditional birth attendants and thirdly on village doctors to treat the neonates. The probable reasons could be their easy availability and provision of medicines upon consultation [6].

The present study found that the male and female guardians and the traditional birth attendants believed that the neonatal complications were outcomes of past sins committed by the parents. Moreover, they thought that these complications occurred when the mothers disobeyed religious rules and customs. Similar findings were indicated in another study conducted in Papua province, Indonesia, which revealed that the child’s ill health was perceived as a cause of parents’ past mistakes, demonic activities, or bad habits, which could result in a delay in decision-making and seeking essential newborn care [23].

The mothers of the neonates were found to have better perceptions regarding neonatal care and complications, although various familial and societal barriers stood in the way of proper practice. These potential barriers could be lack of knowledge of danger signs, non-availability of appropriate health care services, treatment costs, transport expenses, non-accessibility, lack of decision-making power among women, and cultural beliefs and perceptions. In low and middle-income countries, the husband, being the sole bread-winner, is thought to be the household head. Therefore, he is the sole decision-maker regarding seeking newborn care. This gives women very little say in their child’s health and their health [12, 24]. This contradicts the study conducted in Papua province, Indonesia, which stated that women were the family’s principal decision-makers [23]. A study conducted in rural Ghana stated that mothers were often unable to identify the danger signs in their babies and did not seek care at the health facilities even when they realized that their babies were seriously ill [25]. In Bangladesh, almost 87% of the mothers sought care for their newborns, out of which 38% sought care from homoeopaths, 37% from village doctors, 17% from trained providers and 5% from government health facilities [26]. In terms of education and employment, empowerment of women could bring about a considerable improvement in their healthcare decision-making power.

About 53.3% of the mothers underwent delivery at home, where only one-fourth of the deliveries were conducted with three selected newborn practices and clean cord keeping practices, and more than 50% of the cases were found to practice early breastfeeding and delayed bathing [5]. About 39.5% of the caregivers had seen a sick neonate in the family in the last 2 years, among which 30.3% mentioned illness as continuous crying and only 23% of neonates received proper health care. During severe neonatal complications, such as chest in-drawing and rapid breathing, the community people preferred traditional medicines [6]. Most of the mothers were aware that neonatal pneumonia might occur due to exposure to cold. They were also able to identify labour breathing, chest retraction, lethargy and inability to feed as neonatal danger signs which needed outside treatment. The proper health-seeking behaviour of mothers during neonatal complications could play an essential role in preventing child deaths [7]. Breastfeeding should be started within 1 h of birth because delayed breastfeeding might increase the risk of neonatal mortality. A previous study advocated that about 16% of the neonatal deaths could be reduced if breastfeeding was started on the first day of life and 22% if started within the first hour of birth [8].

Community awareness regarding neonatal morbidity and importance of care-seeking from trained providers is an essential step towards reducing neonatal morbidity and mortality [4]. Community neonatal mortality can be effectively reduced by a robust home care strategy to promote an integrated package of preventive and curative newborn care [21]. Only 20–24% sensitivity was found regarding the maternal report of neonatal complications, where 45–54% found optimistic prediction [11]. A local community sensitization program is essential for promoting care-seeking from qualified health care providers during neonatal complications [3]. Community awareness of appropriate health-seeking behaviour and health literacy on the delivery and newborn practices is essential. Bangladesh has already targeted and invested in that direction [4]. The traditional birth attendant (TBA), kaviraj, community health care providers (CHCP), Health Assistant (HA) and Family welfare assistant (FWAs) could be more appropriately utilized in facility-based delivery and for improving newborn care practices in Bangladesh.

Proper perceptions regarding neonatal care are essential to protect the neonates from complications and deaths in the community. Newborn care should include prevention of hypothermia, eye and cord care, early and exclusive breastfeeding, immunization and early identification of danger signs [27]. During neonatal illnesses and complications, the priority should be care-seeking from the qualified providers and proper, timely treatment. Community awareness regarding neonatal complications and practices for care-seeking could bring about considerable improvements in neonatal health. There is an urgent need to strengthen the routine checkup of neonates by health care providers at the community level to eliminate the myths and superstitions and to remove the dependency on traditional healers.

The qualitative research method is an established paradigm of inquiry, where analysis is the most important [13, 14, 28]. A qualitative study using thematic analysis has some merits in the current study context. Here, as a bottom-up approach, we have excerpted themes from the texts instead of categorizing them in a research team. Thematic analysis is useful for summarizing the essential aspects of an extensive qualitative data [14, 28]. Thematic analysis has provided a better option for considering well-structured access for data handling, which helped generate the final results [14, 29]. However, we have not resorted to any theoretical platform such as grounded theory or ethnography or phenomenology. As a flexible method, the thematic analysis could lead us to a lack of coherence for generating themes [28, 29]. To avoid these loophole, senior researchers have controlled the analysis at every stage. We have ensured credibility, transferability, dependability, and confirmability to fulfil the trustworthiness [14, 30].

## Conclusion

Proper perceptions and practices regarding neonatal care and complications are essential steps in the community to strengthen appropriate support of neonates and early care seeking during any neonatal illness. Barriers in the society also influence the provision of proper neonatal care and treatment from qualified healthcare providers to reduce the neonatal deaths in the community in Bangladesh, in the long run. As mentioned earlier, policymakers must focus on these issues for reducing neonatal deaths, which will ultimately result in achieving the Sustainable Development Goals (SDGs) in Bangladesh.

## Supplementary Information

**Additional file 1.**

## Data Availability

Qualitative data available from Dr. Animesh Biswas (Email: ani72001@gmail.com).

## Abbreviations

ARI: Acute respiratory illness
CHCP: Community health care providers
FGD: Focus group discussions
FWA: Family welfare assistant
HA: Health Assistant
IDI: In-depth interviews
MDG: Millenium Development Goal
NMR: Neonatal mortality rate
SDG: Sustainable Development Goals
TBA: Traditional birth attendant
