# Supplementary material for: Perceptions and practices on newborn care and managing complications at rural communities in Bangladesh: a qualitative study
Source: BMC Pediatr. 2021 Apr 9;21:168. doi: 10.1186/s12887-021-02633-z (PMC8033655; doi:10.1186/s12887-021-02633-z)
Supplement: Supplementary file 1 — Additional file 1. [file 12887_2021_2633_MOESM1_ESM.docx]

**Additional files**

**Indepth interviews (IDIs)**

**Regarding the general/basic idea about neonatal complications:**

1. Please tell us what your understanding of neonatal complications is?

2. If you know about this, where did you find out and who informed you?

3. If you don't know, can you please mention why don't you know?

**Regarding the practice of prevention of neonatal complications:**

1. How did your family prepare for the neonatal complications?

2. How was the baby's body cleansed after birth and what measures were taken to keep the baby's body warm?

3. How long after birth was the baby breastfed?

4. How was the navel care taken of the newborn?

5. What arrangements were made for the immediate care of the newborn during delivery?

6. Where and to whom the newborn was taken during complications? Why did you go there?

7. If you were not prepared to prevent complications, why not?

**Regarding the main content of neonatal complications:**

1. The newborn could not or less suck breast milk? What needs to be done in this case?

2. Did the baby's chest drowning/ did he breathe frequently? So what did you do / what do you think should be done?

3. Did the newborn have a fever / did the body suddenly become cold? If so, what did you do?

4. Did the movement of the baby decreased/slow down? So what did you do / what do you think should be done?

5. Whether the newborn was convulsed? So what did you do / what do you think should be done?

6. Whether the baby was just coking (noising)? If so, what did you do?

7. Did the navel turn red / did the pus come out? If so, what did you do?

8. Did the skin of the eyes and skin turn yellow and spread to the hands and feet' palms (Jaundice)? If so, what did you do?

**The necessity to know about the neonatal complications:**

1. Mention what you think the necessity to know about neonatal complications?

2. Why do you think it is essential to know about neonatal complications?

**Obstacles to prevent neonatal complications:**

1. Do you think there were any barriers in the family (father's house/father-in-law's house) and society to cure these neonatal complications? If so, what were the obstacles?

2. How these obstacles can be overcome /removed?

**Regarding the implementation of the practice to prevent neonatal complications:**

1. What steps can be taken to disseminate the necessary information for preventing neonatal complication?

2. How do you think awareness (practice) can be increased to remedy neonatal complications?

**Guideline:** **Focus group discussion (FGD)**

1. General perception on the neonatal complications

**Probe:** basic understanding, Source of knowledge

1. Practice of prevention of neonatal complications

**Probes:** Preparation, Bath after birth, Breastfeeding, navel care, immediate care after birth, essential newborn care, care during complications, seeking treatment

1. Perception and practices on neonatal complications

**Probe**: Less suck breast milk, baby's chest drowning, fever / suddenly become cold, movement of the baby decreased, convulsion, just coking (noising), navel turn red / pus come out, Jaundice

1. Necessity to know about the neonatal complications

**Probe:** Need to know, Importance to know

1. Obstacles to prevent neonatal complications

**Probe:** barriers in the family and society, overcome to obstacles

1. Practice of prevention of neonatal complications

**Probes:** Dissemination of information, awareness to remedy neonatal complications
